# Development of Central Venous Stenosis Upon ICD Implantation in Dialysis Patients: A Non-Negligible Issue

**DOI:** 10.26502/fccm.92920253

**Published:** 2022-04-14

**Authors:** Rohit J Timal, Ioannis Karalis, Jose M Montero Cabezas, Joris I Rotmans, Liselotte C R Hensen, Maurits S. Buiten, Mihaly K de Bie, Lieselot van Erven, Hein Putter, Martin J. Schalij, Ton J Rabelink, J Wouter Jukema

**Affiliations:** 1 Department of Cardiology, Leiden University Medical Center, Leiden, The Netherlands.; 2 Department of Internal Medicine, Leiden University Medical Center, Leiden, The Netherlands.; 3 Department of Biomedical Data Sciences, Leiden University Medical Center, Leiden, The Netherlands.

**Keywords:** Prospective study, Subclavian vein stenosis, Central venous stenosis, Venography, Phlebography, dialysis, End-stage renal disease, Cardiac device complication, Implantable cardioverter-defibrillator, ICD, Cardiac implantable electronic device, Lead-related central vein stenosis

## Abstract

**Background:**

In hemodialysis patients, implantable cardioverter-defibrillator (ICD) implantation may result in central venous stenosis (CVS) with associated symptoms, such as pain, edema of the ipsilateral arm, facial edema, and loss of dialysis access. However, literature concerning CVS in dialysis patients with a cardiac implantable electronic device is scarce.

**Methods:**

We conducted a prospective cohort study in which we investigated the incidence of CVS in end-stage renal disease patients on chronic dialysis who received an ICD as part of participation in the randomized ICD2 trial. A venography was performed before ICD implantation and at 1 year follow-up.

**Results:**

Between 2007 and 2017, 80 patients on dialysis received an ICD according to ICD2 trial protocol. Our population mainly consisted of males (76.3%), and had a median age of 67 years. Hemodialysis was the predominant dialysis modality (71.3%). The ICD was implanted in the right pectoral region in 58 patients (72.5%). A minority of the patients (27.5%) had a history of central venous catheters use, ipsilateral to ICD implantation site. Median follow-up was 16 months (IQR 13–35). Prospective assessment of central vein patency was possible in 56 patients (70.0%). Partial obstruction of central vein at follow-up was present in 19 out of 56 patients (33.9%) and complete occlusion in 4 patients (7.1%). With a complete clinical follow-up of all patients with a median duration of 3.5 years (IQR 2.7 – 6.3), 3 patients developed clinically significant symptoms of CVS.

**Conclusions:**

Development of CVS in patients on chronic dialysis who received an ICD is a cause of concern. Prevention of such complications deserves attention and further research.

**Trial Registration:**

ISRCTN20479861

## 1. Introduction

Implantation of cardiac implantable electronic devices (CIED), such as pacemakers and implantable cardioverter-defibrillators (ICD), can be life-saving as they can restore brady – and tachyarrhythmias [1]. End-stage renal disease patients on dialysis are at high risk for these arrhythmias and the use of CIEDs is not uncommon [2–5]. Although leadless devices are increasingly used, transvenous devices are still the most common method of implantation [6]. However, transvenous cardiac device placement can lead to severe vascular and/or infectious complications, especially in vulnerable populations such as dialysis patients [7,8]. A well-functioning vascular access is crucial in managing patients on hemodialysis. Central venous stenosis (CVS), defined as stenosis in the brachiocephalic, subclavian or superior cava vein is associated with symptoms such as edema and pain in the ipsilateral extremity, and may result, specifically in hemodialysis patients, in a compromised usability of their arteriovenous access [9–12]. Management of central venous occlusive disease (CVOD) consists of (often repeated) percutaneous transluminal angioplasty; in refractory cases, endovascular banding/ligation of the functioning arteriovenous fistula (AVF) or removal of the ICD leads may be necessary [10,13]. Very little is known with regard to development of lead-induced CVS after CIED implantation in patients on dialysis. To the best of our knowledge, our study is the first to prospectively investigate the development of CVS upon a device implantation in dialysis patients. In this report, we aim to clarify the incidence and consequences of lead-induced CVS in this vulnerable patient group.

## 2. Methods

### 2.1 Study design and population

We performed a preplanned subanalysis of The Implantable Cardioverter-Defibrillator in Dialysis Patients (ICD2) trial14. In the randomized, controlled ICD2 trial we demonstrated that prophylactic ICD therapy in patients on chronic dialysis with LVEF ≥35% was not associated with a reduced rate of sudden cardiac death or all-cause death. Patients were referred from 17 dialysis centers to the Leiden University Medical Center, in Leiden, The Netherlands. Patients on dialysis for more than 90 days, with an age of ≥55 years and <81 years, and not meeting the class I indication for ICD implantation were eligible for participation. Exclusion criteria were: heart failure (New York Heart Association functional class IV); medical condition determining a life expectancy <1 year; expected kidney transplantation within 1 year; CVC in situ; patients with acute myocardial infarction in the past 40 days; known human immunodeficiency virus infection and inability to provide informed consent. Patients were randomized in a 1:1 fashion to receive an ICD (ICD group) or standard care (control group). Patients assigned to the ICD arm received a dual-chamber transvenous device (Biotronik, Berlin, Germany), which was implanted subcutaneously in the pectoral region, under local anesthesia. Baseline characteristics, such as patient demographics, comorbidity, and medication use were collected. Dialysis characteristics were extracted from the hospital’s electronic medical records. The protocol was approved by the hospital’s ethics committee in April 2007. The trial is registered at the Netherlands Trial Register (http://www.controlled-trials.com/ISRCTN20479861). All patients provided written informed consent. In October 2012 we added an amendment to the trial protocol that facilitated venography pre-randomization as standard screening procedure, as well as venography at 1-year follow-up in order to prospectively assess CVS in patients undergoing chronic dialysis. Patient enrollment ensued until January 2018. Extended clinical follow-up ensued until the ICD2 trial was stopped in February 2018 per advice of the data and safety monitoring board because of futility.

### 2.2 Venography

Angiographic evaluation was performed by injecting 10ml of iodine-based contrast using a peripheral venous canula, ipsilateral to the ICD-implantation site. In general, systems classifying CVS are based on anatomic location of lesion, degree of occlusion and degree of collateral flow (Supplemental eTable 1). We chose to define phlebographic images according to 2 systems. First, images were adjudicated according to the following 3 categories: completely obstructed, partially obstructed (>70%) or patent. Secondly, the degree of collateral flow was classified as ‘mild’ (residual collateral flow and/or 1 collateral vein); ‘moderate’ (moderate collateral flow with incomplete opacification of the vein proximal to the occlusion and/or 2 collateral veins can be seen) and ‘important’ (complete and rapid collateral flow and/or ≥3 collateral veins). All angiographic images were independently evaluated by two experienced interventional cardiologists (I.K. and J.M.M.C). In case of verdict discrepancy with regard to degree of stenosis or collateral flow, images were assessed by a third experienced interventional cardiologist (J.W.J).

### 2.3 Statistical analysis

Categorical data are presented as absolute numbers (%) and continuous data as mean ± SD, or median and interquartile range (IQR) in case of a non-normal distribution. Using relative risk (RR) statistical differences were tested for occurrence of central vein stenosis and history of ipsilateral CVC use. Statistical significance was tested using the Chi-square test. A p-value below 0.05 was considered statistically significant for this analysis. All statistical analyses were performed using SPSS version 25.0 (IBM Corp, Armonk, New York, USA).

## 3. Results

In total, 80 patients undergoing chronic dialysis received an ICD according to the ICD2 trial protocol. Procedural characteristics such as ICD type, right atrial and ventricular leads have been described previously [14]. All patients received dual-chamber ICDs, with the exception of 2 patients who received a device with a single right ventricular lead because of permanent atrial fibrillation. Baseline characteristics are depicted in table 1. Our population mainly consisted of males (76.3%), with a median age of 67 years, IQR 63–74 years. Hemodialysis was the predominant dialysis modality (71.3%). History of central venous catheters (CVC) ipsilateral to ICD implantation site was present in 22 out of 80 patients (27.5%). ICD was implanted in the right pectoral region in 58 patients (72.5%). Median follow-up was 16 months (IQR 13–35). Angiography at baseline was performed in 62 patients (77.5%). In the remaining 18 patients we considered the ipsilateral central venous circulation patent, as all of these patients underwent successful transvenous ICD implantation. In 7 cases (12.5%) venography images at baseline were not available. In these cases, information regarding the patency status of the central vein was extracted from the procedure report. For the patients where angiography images were available the percentage of agreement among the reviewing cardiologists was high, in more than 95% of the cases. The inter-reviewer reliability is depicted in Supplemental eTable 2.

**Figure 1:**
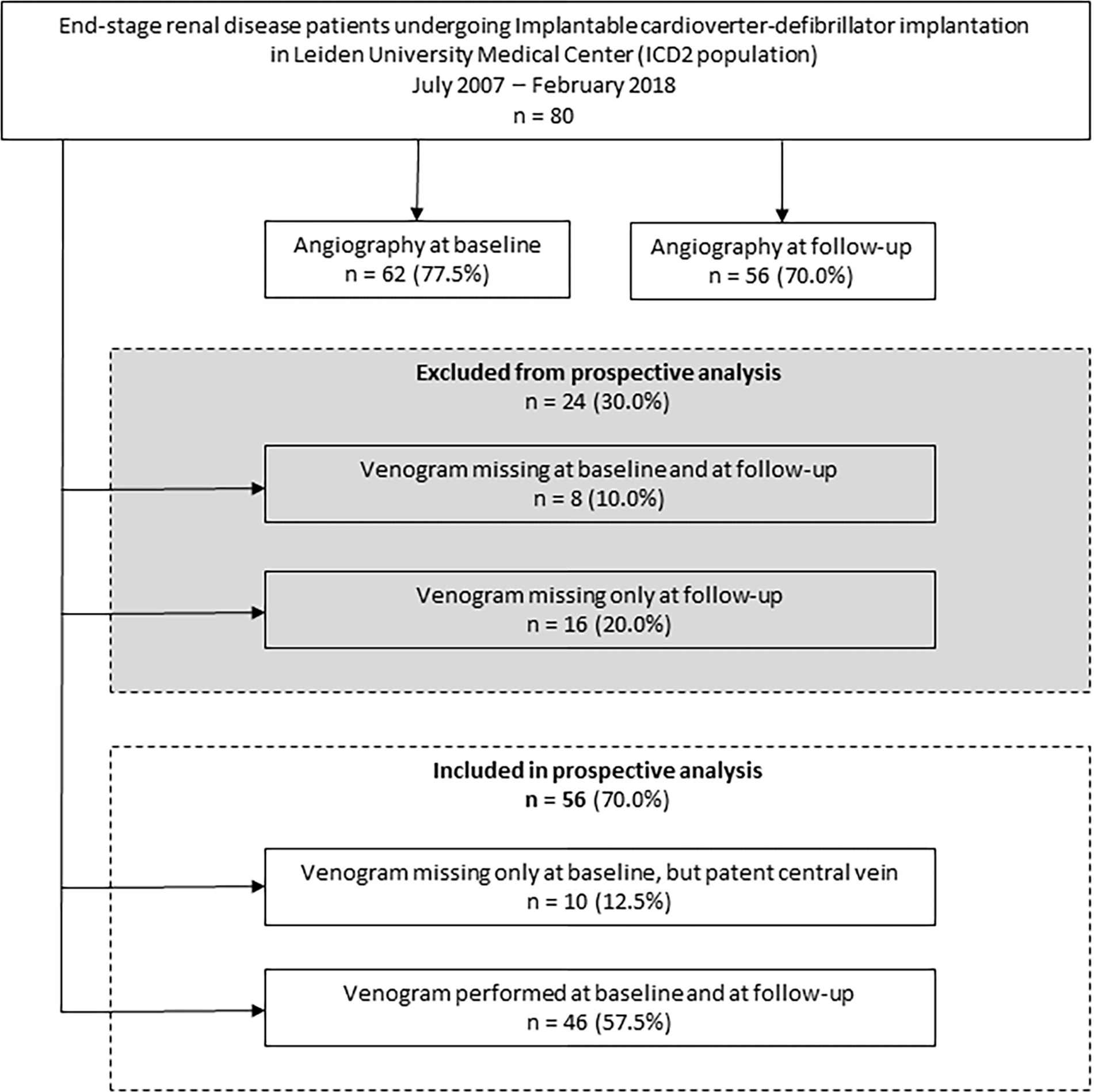
Study flow chart N indicates number.

**Figure 3:**
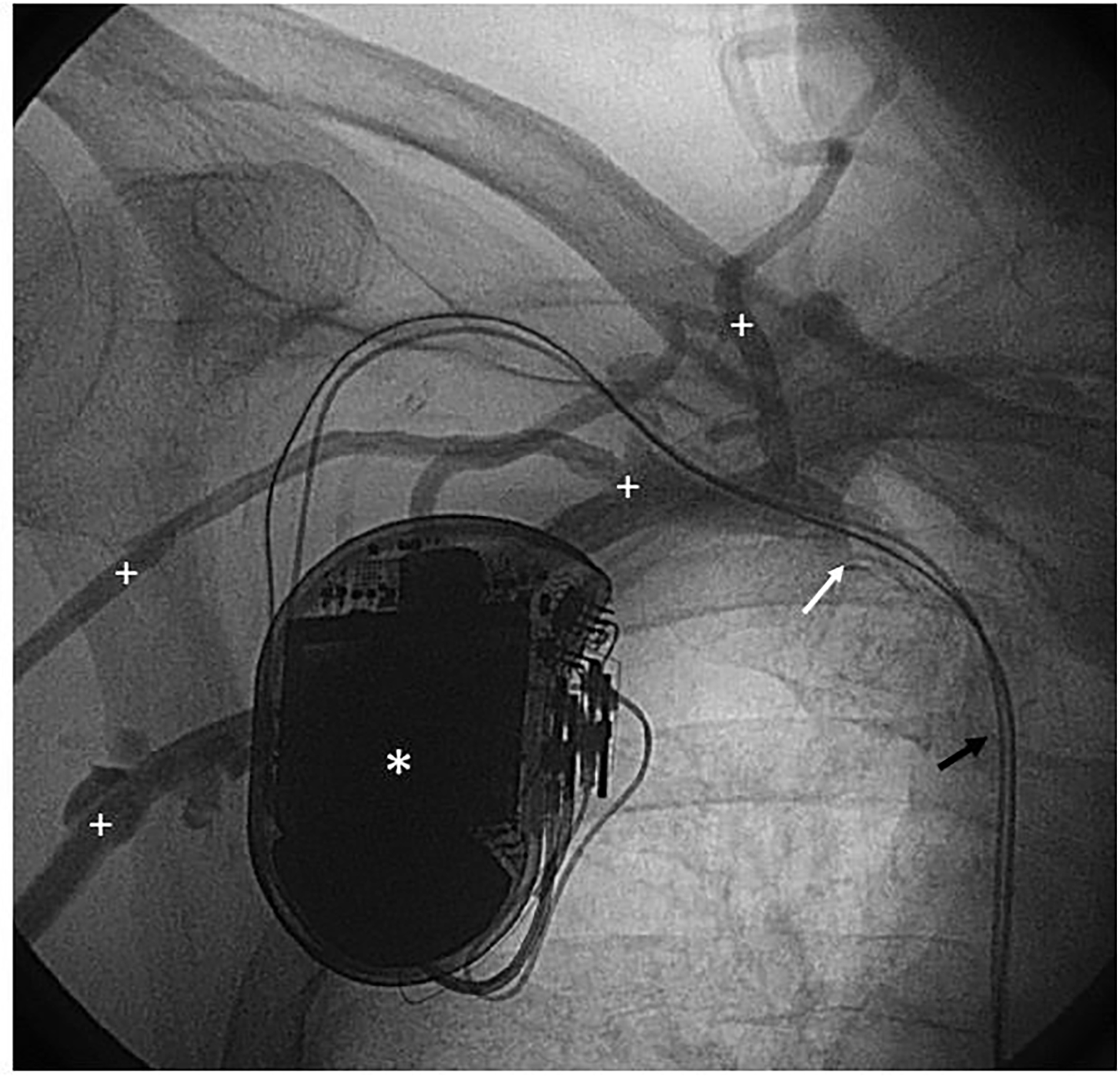
Venography: Patient (ICD 47) undergoing venography at follow-up. * Implantable cardioverter-defibrillator; black arrow: right atrial and right ventricular leads; ^+^ indicates extensive collateral veins; white arrow: central vein stenosis.

Prospective assessment of central vein patency was not possible in 24 patients (30.0%) (Figure 1). Five out of these 24 patients (6.3%) were deceased at 1 year follow-up. The 5-year survival probability from ICD implantation is depicted in Supplemental eFigure 1. The mortality rates at 2, 3, 4 and 5 years were 11 (13.8%), 21 (26.3%), 32 (40.0%), and 33 (41.3%) out of 80, respectively. In 56 out of 80 patients (70.0%) angiography was performed at follow-up and these cases were available for prospective analysis of central vein patency (Table 2 and Table 3). Angiographic progression to CVS occurred in 22 of 56 patients (39.3%), of which 9 patients with a history of CVC use ipsilateral to ICD implantation site (40.9%) versus 13 patients without a history of ipsilateral CVC use (59.1%), relative risk 1.9; 95% confidence interval 1.0 – 3.5; p=0.055. The anatomic location of CVS ipsilateral to ICD implantation site was the subclavian vein in 20 out of 22 patients (90.9%), of which 4 cases with complete stenosis. The brachiocephalic vein was afflicted in 2 out of 22 patients (100% inter-rater reliability). In total, 11 patients with normal baseline venography developed signs of collateral flow. An example of CVS can be seen in figure 2 and figure 3. During clinical follow-up of ICD-recipients with a median duration of 3.5 years (IQR 2.7 – 6.3) from ICD implantation date, 3 patients developed clinically significant symptoms of CVS. These cases are described below.

### ICD57:

ICD was implanted via the right subclavian vein in September 2009. Patient was admitted in November 2009 for right atrial and right ventricular lead repositioning after dislocation in the context of Twiddler syndrome. Four years later, patency of the AVF in the left arm was lost following a traumatic event, after which new AVF was created, ipsilateral to ICD implantation site. Another 4 years later, the patient reported symptoms of pain and edema in the right arm, which appeared to be secondary to venous occlusion. In August and November 2017 percutaneous transluminal angioplasties were performed. Unfortunately, the complaints of pain and edema persisted, whereupon the AVF was ligated.

### ICD 167:

ICD was implanted right-sided in September 2014. At that time, the flow of the ipsilateral AVF was 550mL/min. Patients developed edema of the right arm in August 2015. Visualisation of the subclavian vein in August 2015 revealed a significant stenosis near the transition of the brachiocephalic vein to the superior vena cava which was left untreated as cannulation of the AVF was still possible and symptoms were mild. AVF flow and dialysis efficacy remained stable during follow-up.

### ICD 195:

The ICD was implanted in April 2016 in a patient treated with peritoneal dialysis. Implantation location was the right pectoral region. For clinical reasons, the dialysis modality was changed from peritoneal dialysis to hemodialysis 3 months after ICD implantation. In July 2016, an AVF was created in the right arm. Within the next month, the patient developed edema of the right arm. This was bothersome and it impeded cannulation of the fistula for dialysis. Therefore, hemodialysis was temporarily performed using a CVC. A venogram showed a mild stenosis in the transition from the subclavian vein to the vena cava superior with extensive collateral formation. The AVF had a high flow of >3 L/min, which was responsible for the edema of the arm as this venous stenosis precluded sufficient drainage of the AVF flow to the heart. During a 3-day admission the ICD and leads were removed, and an attempt was made to dilate the venous trajectory on the right-pectoral side, using rotablation and cutting balloon. The procedure was complicated by bleeding in the ICD pocket. Afterwards, a banding procedure of the fistula was performed with the aim to reduce AVF flow, whereupon the edema diminished.

## 4. Discussion

We conducted a cohort study in which we prospectively evaluated the incidence of transvenous-lead-associated CVS by performing angiographic evaluation in 80 patients on chronic dialysis that received a transvenous ICD for primary prevention included in the ICD2 trial [14]. Prospective angiographic assessment of central vein patency, with a median of 16 months from baseline angiography, revealed partial obstruction of the central vein in a third of patients, and a total occlusion of the central vein in 7.1% of patients. During clinical follow-up, with a median duration of 3.5 years from ICD implantation date, 3 patients developed CVS-related symptoms such as pain and edema of the ipsilateral arm. It is noteworthy that in 2 out of these 3 patients with complaints, the symptoms arose after the surgical creation of an AVF while an ipsilateral ICD was already in situ. This underlines the importance of the guideline recommendation that vascular access should be created in the opposite arm [15]. Literature concerning CVOD in dialysis patients with implanted cardiac devices is scarce. This topic has previously been addressed in case reports and in (small) retrospective studies [3,11,16–22]. To the best of our knowledge, our study is the first to prospectively describe the development of CIED lead-induced CVS in patients on chronic dialysis. Importantly, in the current report central vein patency was assessed at 2 timepoints: previous to ICD insertion and at median follow-up of 16 months. Patients were referred from multiple dialysis centers in the Netherlands (n = 15), thus making the results more generalizable. Noteworthy is that, in our trial, ICD placement was performed in patients on dialysis with pre-existing and functioning vascular access of the upper extremity. Furthermore, ICD was implanted contralateral to the dialysis shunt, as was recommended in the literature [3,6,23]. Jeong and colleagues retrospectively investigated 42 patients on hemodialysis, of which 22 patients with vascular access of the upper extremity ipsilateral to transvenous CIED and 20 patients with a dialysis shunt contralateral to CIED19. After an 11 months follow-up period researchers found a CVS incidence of 27% (6 out of 22) in the ipsilateral vascular access group and 5% (1 out of 20) in the contralateral vascular access group. They concluded that transvenous device implantation, ipsilateral to vascular access should be avoided if feasible. In another retrospective study by Saad and coworkers patients, the prevalence of CIEDs was 10.5% among 1235 chronic hemodialysis patients3. Number of central venous interventions in patients with ipsilateral vascular access to CIED placement was 0.59 per access year versus 0.28 per access year in patients with contralateral vascular access, P <0.001. Adwaney et al. retrospectively assessed 2811 patient on hemodialysis, of which 120 patients (4.3%) were radiologically identified to have CVS9. Only 7 patients in this population were reported to have a pacemaker. Because of the design of this study, the CVS incidence is likely to be a considerable underestimation. CVOD in patients on dialysis without cardiac devices has been described extensively and the reported incidence varies from 4 to 41% [9,24–27]. Prevention of CVOD by minimizing exposure to well-established risk factors, such as the (duration of) use CVCs for vascular access, is of the utmost importance [11,28]. CVS after CIED implantation is a well-recognized late complication in patients without end-stage renal disease. The reported incidence varies from 14 to 64% [29–31]. Haghjoo et al. performed contrast venography in 100 non-dialysis patients who were candidates for generator change, lead revision, or device upgrade. Authors found an incidence of venous obstruction of 26% (9% total obstruction, 17% partial obstruction) after 8 ± 4.5 years since lead implantation [29]. Da Costa et al. conducted digital subtraction venography after 6 months following pacemaker implantation among 229 non-dialysis patients [30]. In their study, abnormal venographies were observed in 64% of the patients. In the 2 beforementioned reports baseline venography was not performed. Korkeila et al. prospectively quantified changes by contrast venography at baseline and at 6 months after CIED-implantation in 150 non-dialysis patients [31]. The authors found an incidence of 14% for new obstructive venous lesions. The wide ranges of reported CVOD incidence may be explained by the use of various classification systems for defining CVS in studies evaluating CVOD (Supplemental eTable 1), making results difficult to compare. Some aspects of our study require further discussion. One limitation is the lack of angiographic follow-up in 30.0% of patients. It is noteworthy that only 1 of these patients developed clinical signs of CVS during extended clinical follow-up (see case 57). In the remaining 56 patients with complete angiographic follow-up, which is the largest population published to date, 41.1% showed angiographic signs of CVS. Secondly, in our study, the venographies were assessed by two independent physicians by eyeballing; no quantitative measurements were performed. Nonetheless, the percent agreement of the interrater reliability regarding CVS was >96% (Supplemental eTable 2). The development of leadless cardiac devices, such as subcutaneous ICDs and leadless pacemakers, offers opportunities to avoid lead-associated CVS in the dialysis population [32–36]. Unfortunately, with regard to complications, still little is known from these techniques in this vulnerable patients. In conclusion, prospective angiographic evaluation of central vein patency in 80 patients on chronic dialysis that received a transvenous ICD for primary prevention revealed significant progression of central vein obstruction in >40% of the patients, after a median follow-up of 16 months. Development of lead-associated CVS in ICD-recipients on chronic dialysis is a clear concern. Prevention of complications such as loss of dialysis access is of the utmost importance. Health care providers should strive for future access protection by minimizing CVC use through early referral to surgical access creation. Also, if feasible, efforts should be made to minimize transvenous lead placement. CVS in dialysis patients with CIEDs is an underexposed topic. Further research is warranted for the use of leadless cardiac devices, such as subcutaneous ICDs or leadless pacemakers, in dialysis patients.

## Supplementary Material

Supply

## Figures and Tables

**Figure 2a:**
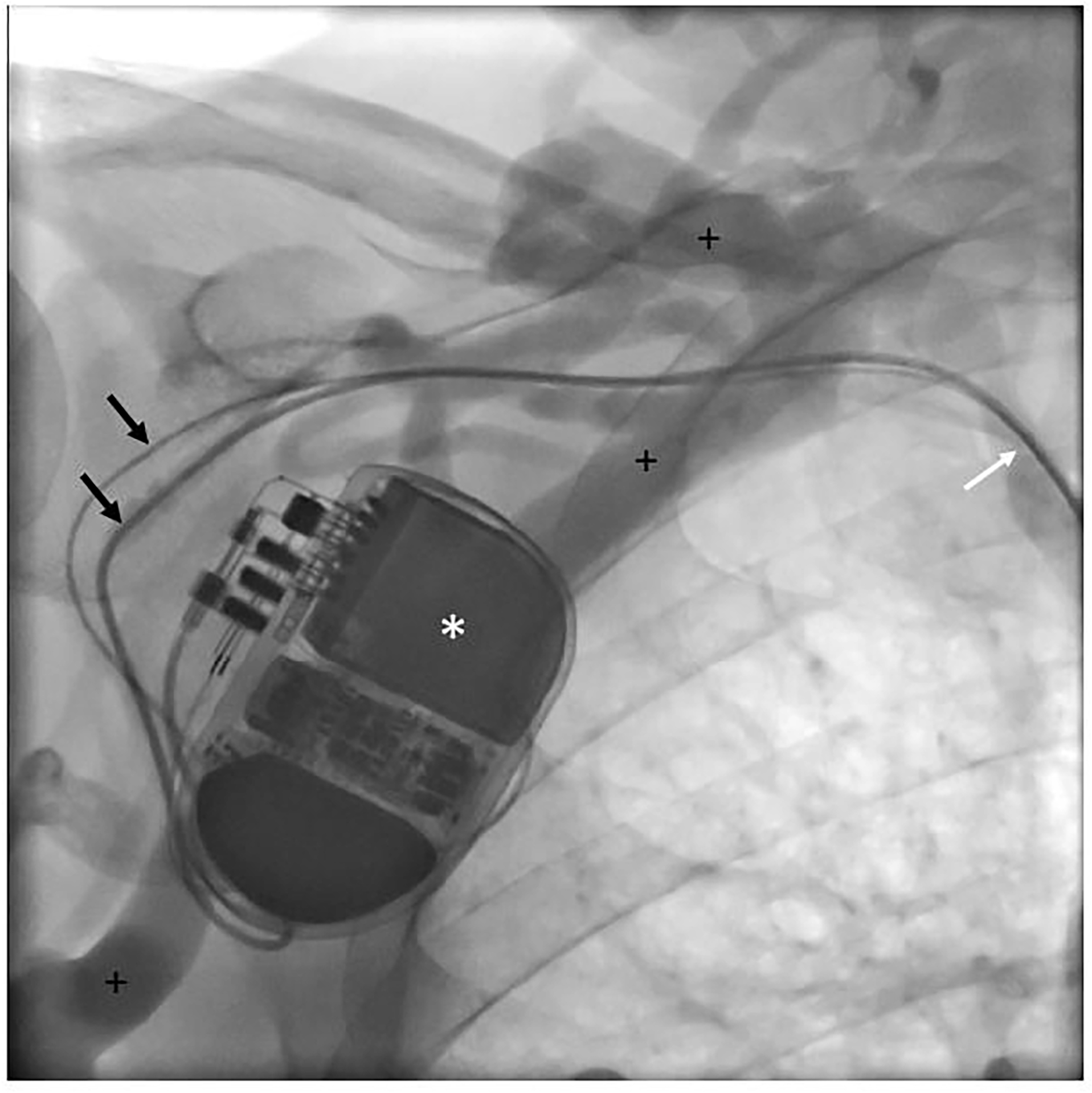
Venography: Patient (ICD 195) undergoing venography at follow-up. * Implantable cardioverter-defibrillator; black arrows: right atrial and right ventricular leads; + indicates extensive collateral veins; white arrow: central vein stenosis.

**Figure 2b:**
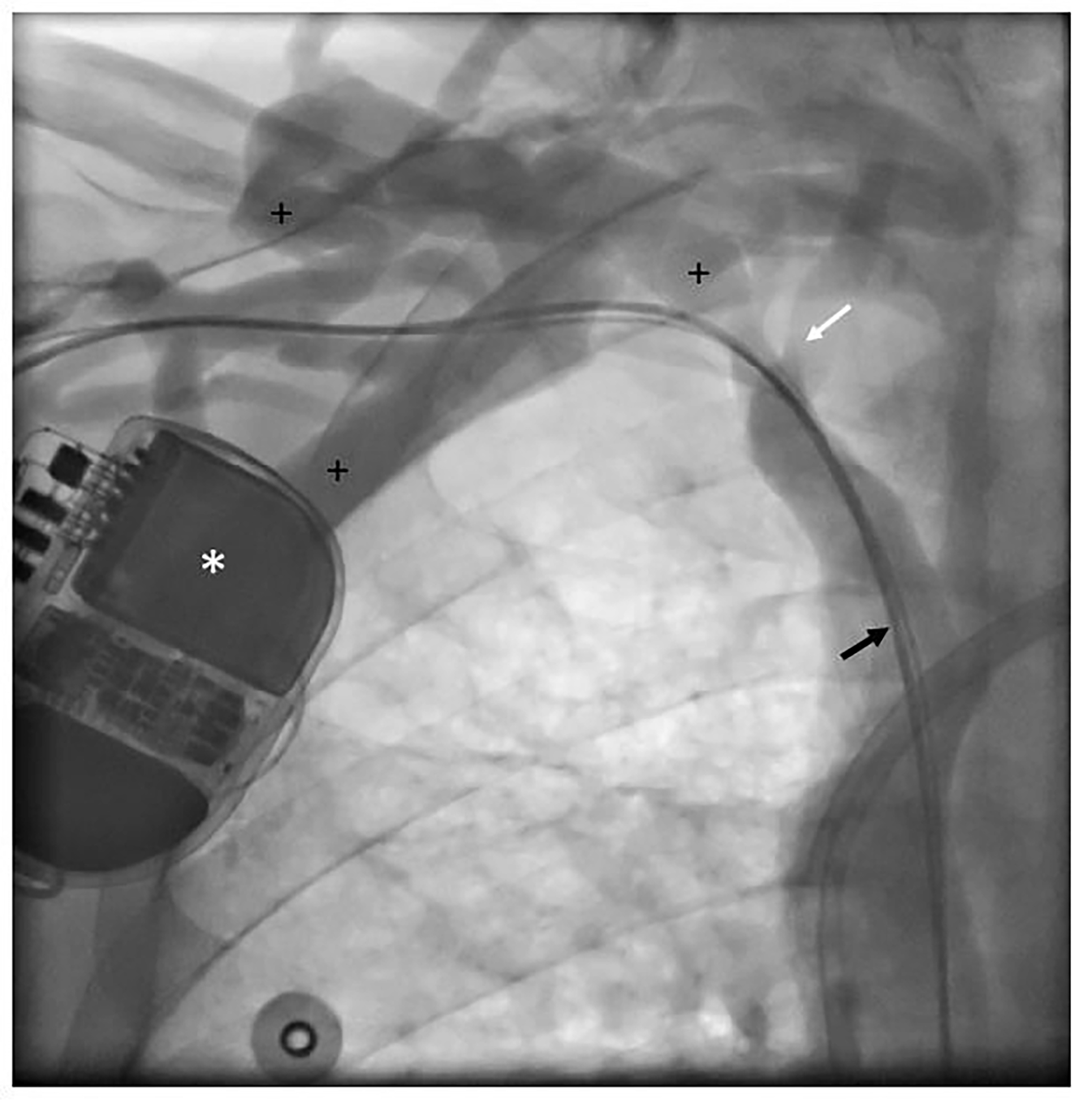
Venography: Patient (ICD 195) undergoing venography at follow-up. * Implantable cardioverter-defibrillator; black arrow: right atrial and right ventricular leads; + indicates extensive collateral veins; white arrow: central vein stenosis.

**Table 1:** Baseline Characteristics

	ICD-recipients on chronic dialysis	Subpopulation with complete prospective assessment
	n = 80	n = 56

Male, n (%)	61 (76.3)	44 (78.6)
Age, years, median (IQR)	67 (63–74)	67 (63–72)
Body mass index, kg/m^2^, mean (SD)	28.2 (5.6)	28.6 (4.8)
Heart rate, bpm, mean (SD)	70 (12)	69 (12)
Blood pressure, mmHg, mean (SD)		
- Systolic	141 (23)	139 (22)
- Diastolic	75 (11)	74 (10)
Dialysis		
Duration of dialysis, months, median (IQR)	16 (9–24)	15 (8–22)
Dialysis modality, n (%)		
- Hemodialysis	57 (71.3)	42 (75.0)
- Peritoneal dialysis	23 (28.7)	14 (25.0)
Vascular access hemodialysis		
- Localization, n (%)		
• Left	43 (53.8)	33 (58.9)
• Right	14 (17.5)	9 (16.1)
- Type, n (%)		
• Goretex graft	4 (5.0)	1 (1.8)
• Brachio-cephalic fistula	23 (28.7)	17 (30.4)
• Cimino fistula	30 (37.5)	24 (42.9)
Kt/V urea/week, median (IQR)		
- Hemodialysis	4.3 (3.6–4.9)	4.3 (3.8–4.9)
- Peritoneal dialysis	2.1 (1.9–2.5)	201 (1.8–2.5)
History of central venous catheter use		
- Ipsilateral of ICD-implantation site, n (%)	22 (27.5)	15 (26.8)
• Short-term (< 14 days)	1 (4.5)	0 (0)
• Mid-term (14 – 90 days)	7 (31.8)	4 (26.7)
• Long-Term (> 90 days)	14 (63.6)	11 (73.3)
• Catheter infections/ Sepsis	2/22 (9.1)	1/15 (6.7)
Medical history, n (%)		
Diabetes mellitus	27 (33.8)	18 (32.1)
Atrial fibrillation or atrial flutter	20 (25.0)	14 (25.0)
Percutaneous transluminal coronary angioplasty	9 (11.3)	6 (10.7)
Coronary artery bypass graft	8 (10.0)	3 (5.4)
Myocardial infarction	16 (20.0)	9 (16.1)
Transient ischemic attack/cerebrovascular accident	13 (16.3)	10 (17.9)
Hypertension	66 (82.5)	46 (82.1)
Hypercholesterolemia	45 (56.3)	32 (57.1)
Malignancy		
- In history	10 (12.5)	9 (16.1)
- Active	2 (2.5)	1 (1.8)
- Radiation therapy in thoracic region	1 (1.3)	1 (1.8)
Smoking, n (%)		
Never	30 (37.4)	22 (39.3)
Yes	17 (21.3)	10 (17.9)
In the past	33 (41.3)	24 (42.9)
Medication use, n (%)		
Vitamin K antagonist	12 (15.0)	9 (16.1)
Platelet aggregation inhibitors	44 (55.0)	29 (51.8)
Cause of end-stage renal disease, n (%)		
Diabetic nephropathy	20 (25.0)	11 (19.6)
Hypertension	27 (33.8)	20 (35.7)
Glomerulonephritis	13 (16.3)	9 (16.1)
Other/unknown	20 (25.0)	16 (28.6)
Laboratory analysis, mean (SD)		
Hemoglobin, mmol/L	7.4 (0.7)	7.3 (0.4)
Hematocrit, %	0.369 (0.035)	0.369 (0.033)
Echocardiography, n (%)		
LVEF (%)		
≥55%	51 (63.7)	36 (64.3)
≥45% and <55%	21 (26.3)	15 (26.8)
≥35% and <45%	8 (10.0)	5 (8.9)
Left ventricular hypertrophy	37 (46.3)	21 (37.5)

N indicates number; ICD, implantable cardioverter-defibrillator; Kt/V, K dialyser clearance of urea; t, dialysis time; V, volume of distribution of urea; LVEF, left ventricular ejection fraction.

**Table 2:** Central vein patency of patients with complete prospective assessment at baseline and at follow-up

	Central Venous Obstruction			
Follow-Up Baseline	Patent	Partial (>70%)	Complete	Total
**Patent, n (%)**	26 (46.4)	17 (30.4)	3 (5.4)	46 (82.1)
**Missing** * **, n (%)**	7 (12.5)	2 (3.6)	1 (1.8)	10 (17.9)
**Total, n (%)**	33 (58.9)	19 (33.9)	4 (7.1)	56 (100)

* Considered patent as ICD was successfully implanted.

N indicates number.

**Table 3:** Collateral flow of patients with complete prospective assessment at baseline and at follow-up

	Collateral Flow				
Follow-Up Baseline	None	Mild	Moderate	Important	Total
**No collateral flow, n (%)**	35 (62.5)	6 (10.7)	1 (1.8)	4 (7.1)	46 (82.1)
**Missing, n (%)**	8 (14.3)	2 (3.6)	0 (0)	0 (0)	10 (17.9)
**Total, n (%)**	43 (76.8)	8 (14.3)	1 (1.8)	4 (7.1)	56 (100.0)

N indicates number.

## References

[1] Al-Khatib SM, Stevenson WG, Ackerman MJ, AHA/ACC/HRS Guideline for Management of Patients With Ventricular Arrhythmias and the Prevention of Sudden Cardiac Death: Executive Summary: A Report of the American College of Cardiology/American Heart Association Task Force on Clinical Practice Guidelines and the Heart Rhythm Society. Heart rhythm (2017).

[2] United States Renal Data System annual data report: Epidemiology of kidney disease in the United States. National Institutes of Health, National Institute of Diabetes and Digestive and Kidney Diseases, Bethesda, (2018).

[3] Saad TF, Ahmed W, Davis K, Cardiovascular implantable electronic devices in hemodialysis patients: prevalence and implications for arteriovenous hemodialysis access interventions. Seminars in dialysis 28 (2015): 94–100. 24863543 10.1111/sdi.12249

[4] Roberts PR, Zachariah D, Morgan JM, Monitoring of arrhythmia and sudden death in a hemodialysis population: The CRASH-ILR Study. PloS one 12 (2017): e0188713. 29240772 10.1371/journal.pone.0188713 PMC5730159

[5] Sakhuja R, Keebler M, Lai TS, Meta-analysis of mortality in dialysis patients with an implantable cardioverter defibrillator. The American journal of cardiology 103 (2009): 735–41. 19231344 10.1016/j.amjcard.2008.11.014

[6] Kusztal M, Nowak K. Cardiac implantable electronic device and vascular access: Strategies to overcome problems. The journal of vascular access 19 (2018): 521–527. 29552930 10.1177/1129729818762981

[7] Buiten MS, DEB MK, VDH AC, Chronic kidney disease and implantable cardioverter defibrillator related complications: 16 years of experience. Journal of cardiovascular electrophysiology 25 (2014): 998–1004. 24758287 10.1111/jce.12435

[8] Polyzos KA, Konstantelias AA, Falagas ME. Risk factors for cardiac implantable electronic device infection: a systematic review and meta-analysis. Europace : European pacing, arrhythmias, and cardiac electrophysiology: journal of the working groups on cardiac pacing, arrhythmias, and cardiac cellular electrophysiology of the European Society of Cardiology 17 (2015): 767–777. 10.1093/europace/euv053 25926473

[9] Adwaney A, Lim C, Blakey S, Central Venous Stenosis, Access Outcome and Survival in Patients undergoing Maintenance Hemodialysis. Clinical journal of the American Society of Nephrology: CJASN (2019). 10.2215/CJN.07010618 PMC6419278 30765534

[10] Agarwal AK. Endovascular interventions for central vein stenosis. Kidney research and clinical practice 34 (2015): 228–232. 26779426 10.1016/j.krcp.2015.10.005 PMC4688584

[11] Chuang CL, Tarng DC, Yang WC, An occult cause of arteriovenous access failure: central vein stenosis from permanent pacemaker wire. Report of three cases and review of the literature. American journal of nephrology 21 (2001): 406–409. 11684804 10.1159/000046284

[12] Teruya TH, Abou-Zamzam AM Jr., Limm W, Symptomatic subclavian vein stenosis and occlusion in hemodialysis patients with transvenous pacemakers. Annals of vascular surgery 17 (2003): 526–529. 12958674 10.1007/s10016-003-0048-4

[13] Asif A, Salman L, Carrillo RG, Patency rates for angioplasty in the treatment of pacemaker-induced central venous stenosis in hemodialysis patients: results of a multi-center study. Seminars in dialysis 22 (2009): 671–676. 19799756 10.1111/j.1525-139X.2009.00636.x

[14] Jukema JW, Timal RJ, Rotmans JI, Prophylactic Use of Implantable Cardioverter-Defibrillators in the Prevention of Sudden Cardiac Death in Dialysis Patients. Circulation 139 (2019): 2628–2638. 30882234 10.1161/CIRCULATIONAHA.119.039818

[15] Schmidli J, Widmer MK, Basile C, Editor’s Choice - Vascular Access: 2018 Clinical Practice Guidelines of the European Society for Vascular Surgery (ESVS). European journal of vascular and endovascular surgery : the official journal of the European Society for Vascular Surgery 55 (2018): 757–818. 10.1016/j.ejvs.2018.02.001 29730128

[16] Drew DA, Meyer KB, Weiner DE. Transvenous cardiac device wires and vascular access in hemodialysis patients. American journal of kidney diseases : the official journal of the National Kidney Foundation 58 (2011): 494–496. 21664734 10.1053/j.ajkd.2011.05.005

[17] Dhamija RK, Tan H, Philbin E, Subcutaneous implantable cardioverter defibrillator for dialysis patients: a strategy to reduce central vein stenoses and infections. American journal of kidney diseases: the official journal of the National Kidney Foundation 66 (2015): 154–158. 25911316 10.1053/j.ajkd.2015.01.028

[18] Saad TF, Hentschel DM, Koplan B, Cardiovascular implantable electronic device leads in CKD and ESRD patients: review and recommendations for practice. Seminars in dialysis 26 (2013): 114–1123. 22891983 10.1111/j.1525-139X.2012.01103.x

[19] Jeong S, Nam GB, Chang JW, Impact of transvenous cardiac implantable electronic devices in chronic hemodialysis patients: a single-center, observational comparative study. BMC nephrology 19 (2018): 281. 30342493 10.1186/s12882-018-1095-y PMC6195973

[20] Deighan CJ, McLaughlin KJ, Simpson K, Unsuspected subclavian vein stenosis resulting from a permanent pacing wire. Nephrology, dialysis, transplantation: official publication of the European Dialysis and Transplant Association - European Renal Association 11 (1996): 2333–2344. 8941605 10.1093/oxfordjournals.ndt.a027163

[21] Korzets A, Chagnac A, Ori Y, Subclavian vein stenosis, permanent cardiac pacemakers and the haemodialysed patient. Nephron 58 (1991): 103–105. 1857465 10.1159/000186387

[22] Tourret J, Cluzel P, Tostivint I, Central venous stenosis as a complication of ipsilateral haemodialysis fistula and pacemaker. Nephrology, dialysis, transplantation : official publication of the European Dialysis and Transplant Association - European Renal Association 20 (2005): 997–1001. 15831549 10.1093/ndt/gfh628

[23] NKF-K/DOQI Clinical Practice Guidelines for Vascular Access: update 2000. American journal of kidney diseases: the official journal of the National Kidney Foundation 37 (2001): S137–81. 10.1016/s0272-6386(01)70007-8 11229969

[24] Park HS, Choi J, Baik JH. Central venous disease in hemodialysis patients. Kidney research and clinical practice 38 (2019): 309–317. 31387161 10.23876/j.krcp.19.025 PMC6727898

[25] Barrett N, Spencer S, McIvor J, Subclavian stenosis: a major complication of subclavian dialysis catheters. Nephrology, dialysis, transplantation: official publication of the European Dialysis and Transplant Association - European Renal Association (1988) 3: 423–425. 3140128 10.1093/oxfordjournals.ndt.a091691

[26] Agarwal AK, Patel BM, Haddad NJ. Central vein stenosis: a nephrologist’s perspective. Seminars in dialysis 20 (2007): 53–62. 17244123 10.1111/j.1525-139X.2007.00242.x

[27] MacRae JM, Ahmed A, Johnson N, Central vein stenosis: a common problem in patients on hemodialysis. ASAIO journal (American Society for Artificial Internal Organs 51 (1992): 77–81. 10.1097/01.mat.0000151921.95165.1e 15745139

[28] Agarwal AK. Central vein stenosis. American journal of kidney diseases : the official journal of the National Kidney Foundation 61 (2013): 1001–1015. 23291234 10.1053/j.ajkd.2012.10.024

[29] Haghjoo M, Nikoo MH, Fazelifar AF, Predictors of venous obstruction following pacemaker or implantable cardioverter-defibrillator implantation: a contrast venographic study on 100 patients admitted for generator change, lead revision, or device upgrade. Europace: European pacing, arrhythmias, and cardiac electrophysiology : journal of the working groups on cardiac pacing, arrhythmias, and cardiac cellular electrophysiology of the European Society of Cardiology 9 (2007): 328–32. 10.1093/europace/eum019 17369270

[30] Da Costa SS, Scalabrini Neto A, Costa R, Incidence and risk factors of upper extremity deep vein lesions after permanent transvenous pacemaker implant: a 6-month follow-up prospective study. Pacing and clinical electrophysiology: PACE 25 (2002): 1301–1306. 12380764 10.1046/j.1460-9592.2002.01301.x

[31] Korkeila P, Nyman K, Ylitalo A, Venous obstruction after pacemaker implantation. Pacing and clinical electrophysiology: PACE 30 (2007): 199–206. 17338716 10.1111/j.1540-8159.2007.00650.x

[32] Mondesert B, Dubuc M, Khairy P, Combination of a leadless pacemaker and subcutaneous defibrillator: First in-human report. HeartRhythm Case Rep 1 (2015): 469–471. 28491609 10.1016/j.hrcr.2015.07.009 PMC5419724

[33] Koman E, Gupta A, Subzposh F, Outcomes of subcutaneous implantable cardioverter-defibrillator implantation in patients on hemodialysis. Journal of interventional cardiac electrophysiology : an international journal of arrhythmias and pacing 45 (2016): 219–223. 26768264 10.1007/s10840-015-0093-2

[34] El-Chami MF, Levy M, Kelli HM, Outcome of Subcutaneous Implantable Cardioverter Defibrillator Implantation in Patients with End-Stage Renal Disease on Dialysis. Journal of cardiovascular electrophysiology 26 (2015): 900–904. 25952566 10.1111/jce.12705

[35] Bardy GH, Smith WM, Hood MA, An entirely subcutaneous implantable cardioverter-defibrillator. The New England journal of medicine 363 (2010): 36–44. 20463331 10.1056/NEJMoa0909545

[36] Maradey JA, Jao GT, Vachharajani TJ. Leadless pacemaker placement in a patient with chronic kidney disease: A strategy to preserve central veins. Hemodialysis international International Symposium on Home Hemodialysis 22 (2018): E57–E59. 29697182 10.1111/hdi.12665

