# Supplementary material for: Development of Central Venous Stenosis Upon ICD Implantation in Dialysis Patients: A Non-Negligible Issue
Source: Cardiol Cardiovasc Med. Author manuscript; Available in PMC 2022 Aug 24. (PMC9401088; doi:10.26502/fccm.92920253)
Supplement: Supply [file NIHMS1823956-supplement-Supply.docx]

**Supplement**

| **Classification systems for central venous stenosis / vein patency** |  |  |
| --- | --- | --- |
| **Anatomic region.** |  |  |
| Stenosis in   - Superior vena cava - Brachiocephalic - Subclavian vein | Asif, Salman et al. 2009^1^ | |
| **Degree of central venous stenosis** |  | |
| - New stenosis: 50% diameter reduction in a venous segment when compared to baseline | Korkeila, Nyman et al. 2007^2^ | |
| - Mild; ≤20% - Moderate; 21% to 69% - Severe; 70% to 99% - Occlusion; 100% | Da Costa, Scalabrini Neto et al. 2002^3^ | |
| - Patent - Partially obstructed (>70%) - Completely obstructed | Haghjoo, Nikoo et al. 2007^4^ | |
| - Patent - Mild degree obstruction (50–75% narrowing) - Severe obstruction (≥75% narrowing) - Total obstruction | Bulur, Vural et al. 2010^5^ | |
| - No stenosis; <50% - Mild stenosis; 50% to 74% - Moderate stenosis; 75% to 89% - Severe stenosis; 90% to 99% - Total occlusion | Sticherling, Chough et al. 2001^6^ | |
| - 50% or more narrowing of the central veins of the thorax that included the superior vena cava, brachiocephalic vein, subclavian vein, and subclavian-cephalic vein junction. | MacRae, Ahmed et al. 2005^7^ | |
| **Subclavian vein patency** |  | |
| - Normal - Equivocal - Abnormal | Barrett, Spencer et al. 1988^8^ | |
| - Normal - Abnormal; >30% narrowing of the vessel lumen diameter, with or without collateral flow | Hernandez, Diaz et al. 1998^9^ | |
| **Degree of collateral circulation** |  | |
| - Mild; 1 vessel, lower flow - Moderate; 2 vessels, moderate flow - Important; ≥ 3 vessels, adequate flow | Da Costa, Scalabrini Neto et al. 2002^3^ | |

## Supplemental eTable 1. Classification systems used in literature

| **Interrater reliability** | Observations (n) | Agreement (n) | Percent  agreement | Kappa | P |
| --- | --- | --- | --- | --- | --- |
| **Baseline** |  |  |  |  |  |
| Central Venous Stenosis | 48 | 47 | 97.9 | n.a.* | n.a.* |
| Collateral Circulation | 48 | 46 | 95.8 | 0.32 (0.16) | 0.000 |
| **Follow-up** |  |  |  |  |  |
| Central Venous Stenosis | 55 | 53 | 96.4 | 0.93 (0.05) | 0.000 |
| Collateral Circulation | 55 | 48 | 87.3 | 0.66 (0.09) | 0.000 |

## Supplemental eTable 2. Interrater reliability

N.a. not applicable. *Kappa could not be calculated because rater 1 had rated all images as "patent".


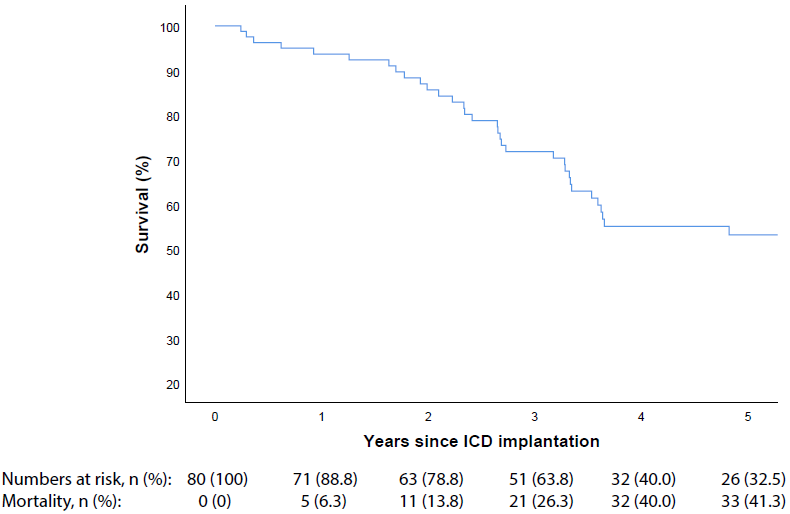


## Supplemental eFigure 1. Kaplan-Meier plot of survival since ICD implantation

Note: Supplemental Figure 1 differs from Supplemental Figure 6. Kaplan–Meier curves for survival per treatment group (PP population), published previously online in Circulation^10^, as Supplemental Figure 1 shows survival from ICD implantation and Supplemental Figure 6 depicts survival from randomization.

# References

1. Asif A, Salman L, Carrillo RG, et al. Patency rates for angioplasty in the treatment of pacemaker-induced central venous stenosis in hemodialysis patients: results of a multi-center study. Seminars in dialysis 2009;22:671-6.

2. Korkeila P, Nyman K, Ylitalo A, et al. Venous obstruction after pacemaker implantation. Pacing and clinical electrophysiology : PACE 2007;30:199-206.

3. Da Costa SS, Scalabrini Neto A, Costa R, Caldas JG, Martinelli Filho M. Incidence and risk factors of upper extremity deep vein lesions after permanent transvenous pacemaker implant: a 6-month follow-up prospective study. Pacing and clinical electrophysiology : PACE 2002;25:1301-6.

4. Haghjoo M, Nikoo MH, Fazelifar AF, Alizadeh A, Emkanjoo Z, Sadr-Ameli MA. Predictors of venous obstruction following pacemaker or implantable cardioverter-defibrillator implantation: a contrast venographic study on 100 patients admitted for generator change, lead revision, or device upgrade. Europace : European pacing, arrhythmias, and cardiac electrophysiology : journal of the working groups on cardiac pacing, arrhythmias, and cardiac cellular electrophysiology of the European Society of Cardiology 2007;9:328-32.

5. Bulur S, Vural A, Yazici M, Ertas G, Ozhan H, Ural D. Incidence and predictors of subclavian vein obstruction following biventricular device implantation. Journal of interventional cardiac electrophysiology : an international journal of arrhythmias and pacing 2010;29:199-202.

6. Sticherling C, Chough SP, Baker RL, et al. Prevalence of central venous occlusion in patients with chronic defibrillator leads. American heart journal 2001;141:813-6.

7. MacRae JM, Ahmed A, Johnson N, Levin A, Kiaii M. Central vein stenosis: a common problem in patients on hemodialysis. ASAIO journal (American Society for Artificial Internal Organs : 1992) 2005;51:77-81.

8. Barrett N, Spencer S, McIvor J, Brown EA. Subclavian stenosis: a major complication of subclavian dialysis catheters. Nephrology, dialysis, transplantation : official publication of the European Dialysis and Transplant Association - European Renal Association 1988;3:423-5.

9. Hernandez D, Diaz F, Rufino M, et al. Subclavian vascular access stenosis in dialysis patients: natural history and risk factors. Journal of the American Society of Nephrology : JASN 1998;9:1507-10.

10. Jukema JW, Timal RJ, Rotmans JI, et al. Prophylactic Use of Implantable Cardioverter-Defibrillators in the Prevention of Sudden Cardiac Death in Dialysis Patients. Circulation 2019;139:2628-38.
